# The Emission Mechanism of Gold Nanoclusters Capped with 11-Mercaptoundecanoic Acid, and the Detection of Methanol in Adulterated Wine Model

**DOI:** 10.3390/ma14216342

**Published:** 2021-10-23

**Authors:** Ming Wei, Ye Tian, Lijun Wang, Yuankai Hong, Dan Luo, Yinlin Sha

**Affiliations:** 1 Single-Molecule and Nanobiology Laboratory, Department of Biophysics, School of Basic Medical Sciences, Peking University, Beijing 100191, China; weiming@bjmu.edu.cn (M.W.); ye.tian1@ge.com (Y.T.); lijunwang@bjmu.edu.cn (L.W.); 2 CAS Center for Excellence in Nanoscience, Beijing Key Laboratory of Micro-Nano Energy and Sensor, Beijing Institute of Nanoenergy and Nanosystems, Chinese Academy of Sciences, Beijing 100083, China

**Keywords:** gold nanocluster, source of absorption, emission mechanism, ligand, size effect

## Abstract

The absorption and emission mechanisms of gold nanoclusters (AuNCs) have yet to be understood. In this article, 11-Mercaptoundecanoic acid (MUA) capped AuNCs (AuNC@MUA) were synthesized using the chemical etching method. Compared with MUA, AuNC@MUA had three obvious absorption peaks at 280 nm, 360 nm, and 390 nm; its photoluminescence excitation (PLE) peak and photoluminescence (PL) peak were located at 285 nm and 600 nm, respectively. The AuNC@MUA was hardly emissive when 360 nm and 390 nm were chosen as excitation wavelengths. The extremely large stokes-shift (>300 nm), and the mismatch between the excitation peaks and absorption peaks of AuNC@MUA, make it a particularly suitable model for studying the emission mechanism. When the ligands were partially removed by a small amount of sodium hypochlorite (NaClO) solution, the absorption peak showed a remarkable rise at 288 nm and declines at 360 nm and 390 nm. These experimental results illustrated that the absorption peak at 288 nm was mainly from metal-to-metal charge transfer (MMCT), while the absorption peaks at 360 nm and 390 nm were mainly from ligand-to-metal charge transfer (LMCT). The PLE peak coincided with the former absorption peak, which implied that the emission of the AuNC@MUA was originally from MMCT. It was also interesting that the emission mechanism could be switched to LMCT from MMCT by decreasing the size of the nanoclusters using 16-mercaptohexadecanoic acid (MHA), which possesses a stronger etching ability. Moreover, due to the different PL intensities of AuNC@MUA in methanol, ethanol, and water, it has been successfully applied in detecting methanol in adulterated wine models (methanol-ethanol-water mixtures).

## 1. Introduction

Luminescent gold nanoclusters (AuNCs) have attracted a lot of attention in recent years due to their good biocompatibility, low biotoxicity, large stokes-shift, long photoluminescence (PL) lifetime, and large two-photon absorption cross-section [1,2,3,4]. Although rapid progress has been made in terms of synthesis and application [5,6,7], the emission mechanism for AuNCs still remains unsolved. The first study on the PL mechanism of bulk gold can be dated back to 1969 by Mooradian [8]. The quantized transitions observed were attributed to interband (d-sp) transitions, and the emission was attributed to a direct radiative recombination of the excited electrons with holes in the d-band. PL from AuNCs was observed after about 30 years in 1998 [9], when the mechanism was intensively studied. In 2001, Huang and Murray [10] suggested that the emission mechanism of four water-soluble monolayer-protected AuNCs was the same as that for bulk gold. However, in 2002, Link et al. [11] suggested that the infrared luminescence of Au_28_SG_16_ was the relaxed radiative recombination within the sp conduction band (intraband transition); they theorized that the emission wavelength of AuNCs was dependent on the number of gold atoms, which decided the energy-level gaps within an sp conduction band. Subsequently, in 2004, Zheng et al. [12] used dendrimers as templates to encapsulate AuNCs, the luminescent properties of which supported Link’s theory. In addition, Au_8_ synthesized with different ligands had the same emission characteristics, which added to the evidence supporting Link’s theory [13,14]. In contrast, in 2005, Negishi et al. [15] synthesized and separated nine AuNCs protected by glutathione: Au_10_(SG)_10_, Au_15_(SG)_13_, Au_18_(SG)_14_, Au_22_(SG)_16_, Au_22_(SG)_17_, Au_25_(SG)_18_, Au_29_(SG)_20_, Au_33_(SG)_22_, and Au_39_(SG)_24_. Their emission properties did not follow Link’s theory; for instance, Au_22_(SG)_16_ and Au_22_(SG)_17_ had the same number of gold atoms, but their emission maxima were not the same. In addition, the maximum emission wavelengths of Au_22_(SG)_16_ and Au_22_(SG)_17_ were blue-shifted, compared with Au_18_(SG)_14_, and Au_29_(SG)_20_, Au_33_(SG)_22_, and Au_39_(SG)_24_ which had the same maximum emission wavelengths, despite having different numbers of gold atoms. Furthermore, Au_25_ synthesized by Negishi et al. ($\lambda_{\max}^{\mathrm{em}}$ = 827 nm) [15], Muhammed et al. ($\lambda_{\max}^{\mathrm{em}}$ = 700 nm) [14], and Xie et al. ($\lambda_{\max}^{\mathrm{em}}$ = 640 nm) [16] had different maximum emission wavelengths. Especially, when N_Au_ > 55 (>1.2 nm in diameter), they were still luminescent [17,18,19], though they were not luminescent through radiative recombination within the sp conduction band [20]. For the N_Au_ > 55 situation, Huang et al. [21] suggested that the emission was derived from metal-centered transitions and/or ligand-to-metal charge transfer (LMCT; S→Au), proposed in Au(I)–ligand complexes [22,23]. Other studies also showed that the charge state and the ligands played important roles in the luminescence of AuNCs [19,24,25]. In 2012, Zheng divided AuNCs into few-atom nanoclusters and few-nanometer nanoclusters [26]; the luminescence mechanism of the former was revealed partially [27,28]. Nevertheless, the sources of emission for the latter were still difficult to attribute clearly.

In this article, we focused on few-nanometer AuNCs. The emission mechanism was partially elucidated by studies of AuNC@MUA. It can also be switched to LMCT from metal-to-metal charge transfer (MMCT) by decreasing the size of the nanoclusters using MHA, which possesses a stronger etching ability. In addition, due to the different PL intensities in methanol, ethanol, and water, AuNC@MUA was applied in detecting methanol in adulterated wine models (methanol-ethanol-water mixtures).

## 2. Materials and Methods

### 2.1. The Chemicals

All chemicals were used directly without further purification: tetrachloroauric acid (HAuCl_4_·4H_2_O, >99.95%) and sodium borohydride (NaBH_4_, ≥98%) were from Aladdin Reagent Co. (Shanghai, China); 11-mercaptoundecanoic acid (MUA, 95%) was from J & K Co. (Beijing, China); 16-mercaptohexadecanoic acid (MHA, 90%) was purchased from Sigma–Aldrich (Shanghai, China); sodium hypochlorite solution (NaClO, available chlorine ≥ 8.0%) was from Xilong Chemical Co. Ltd. (Shantou, China); methanol (≥99.5%) and ethanol (≥99.5%) were from Beijing Chemical Co. (Beijing, China), and ultrapure water (>18 MΩ·cm) was used in the experiments.

### 2.2. Synthesis of Small Gold Nanoparticles Capped by MUA (AuNP@MUA)

The method of synthesizing the AuNP@MUA was analogous to those previously reported [11,15]: 23.0 mg (0.1 mmoL) MUA was added to a methanol solution of HAuCl_4_·4H_2_O (5 mL, 5 mM) in a round-bottom flask and stirred for half an hour in a 0 °C cool bath. Then, 1.25 mL of NaBH_4_ (0.16 M) aqueous solution at 0 ℃ was rapidly added to the mixture under vigorous stirring and the reaction continued for another hour (HAuCl_4_·4H_2_O:MUA:NaBH_4_ = 1:4:8, molar ratio). After the reaction, the by-products and starting materials in the solution were removed by centrifugal filtration (7500× *g*) for 30 min each time, through a filter with a cutoff of 3 kDa. The purified AuNP@MUA solution was obtained after three processes of centrifugal filtration.

### 2.3. Synthesis of Luminescent AuNC@MUA

The all-purified AuNP@MUA solution was diluted to a 12 mL aqueous solution in a round-bottom flask. Then, 23.0 mg MUA and 100 μL NaOH aqueous solution (1 M) were added to the AuNP@MUA solution and isolated from the air by a bottle stopper. The temperature of the mixture was maintained at 55 °C with an oil bath and gentle stirring. The reaction was sustained for 26 h. The luminescent AuNC@MUA solution was purified using the same procedure as for the AuNP@MUA. The AuNC@MUA light brown powder was obtained by lyophilization.

### 2.4. AuNC@MUA Was Treated by NaClO Solution (AuNC@MUA_NaClO_)

A total of 1 mg of AuNC@MUA was diluted in 2 mL of ultrapure water, and 5 μL of the NaClO aqueous solution was added, stirring for 30 min at room temperature. Then, the AuNC@MUA_NaClO_ was purified using the same procedure as for the AuNP@MUA.

### 2.5. Synthesis of Luminescent AuNC@MHA

The synthesis of the AuNC@MHA was similar to that of the AuNC@MUA. The differences were that 32.0 mg of MHA and 100 μL of NaOH aqueous solution (1 M) were added, and the reaction was sustained for 10 h. Then, the luminescent AuNC@MHA solution was purified using the same procedure as for the AuNP@MUA.

### 2.6. Synthesis of Luminescent AuNC@MHA Intermedium (AuNC@MHA_im_)

The synthesis of the AuNC@MHA_im_ was similar to that of the AuNC@MUA. The differences were that 8.0 mg of MHA and 25 μL of NaOH aqueous solution (1 M) were added to the AuNP@MUA solution, and the reaction was sustained for 10 h. The AuNC@MHA_im_ was purified using the same procedure as for the AuNP@MUA.

### 2.7. Assay of Methanol in Adulterated Wine Model (Methanol-Ethanol-Water Mixtures)

In this work, we used a methanol-ethanol-water mixture as a simplified adulterated wine model. A series of concentrations of methanol in methanol-ethanol-water mixtures (V_methanol_ + V_ethanol_ = 500 μL) were added to the AuNC@MUA solutions (500 μL) one by one and equilibrated at 25 °C for 5 min prior to the measurements of the PL with an excitation wavelength at 280 nm. This process was repeated three times.

### 2.8. Instruments and Analysis

The UV–Vis absorbance spectra were acquired with a TU-1901 UV–Vis spectrophotometer (Beijing Puxi Tongyong Co., Beijing, China). The PL and photoluminescence excitation (PLE) spectra were recorded on an F-4500 fluorescence spectrophotometer (Hitachi, Japan). The quantum yield (QY) was measured using rhodamine 6G (Aladdin Reagent Co., Shanghai, China) (QY = 0.95 in ethanol) as the reference. The data for a given sample, with a given excitation wavelength but with different concentrations, should fit nicely in a linear line that must go through the origin. The measurements were conducted at a temperature of 20 °C, and the PL excitation and absorption wavelengths were set at 280 nm for the AuNC@MUA and at 470 nm for the rhodamine 6G. The PL integral area was calculated by the F-4500 fluorescence spectrophotometer software. We performed this by clicking on the area, then setting the wavelength start at the maximum emission wavelength (around 600 nm, avoiding light source scattering, centered at 560 nm), and ending it at 700 nm, to calculate half of the PL integral area of the AuNC@MUA. We then set the wavelength start at 500 nm and end at 700 nm, for calculating the entire PL integral area of rhodamine 6G. The entire PL integral area of AuNC@MUA needed to be multiplied by 2.

Photoluminescence lifetimes were performed on an Edinburgh Instrument lifetime and steady state spectrometer FLS920 (Edinburgh Instruments, UK) with a pulsed light-emitting diode (LED) (280 nm, 40 KHz, 7 V) as the excitation source. The Fourier transform infrared (FTIR) spectra were acquired on a NEXUS-470 FTIR spectrometer (Nicolet Instruments, USA) using KBr pellets ranging from 4000 to 400 cm^−1^. The hydrodynamic diameters of the AuNC@MUA were measured at 25 °C on a ZetaPlus Potential Analyzers (Brookhaven, USA) in ultrapure water at a concentration of 5 μM. An XPS analysis was conducted with an Axis Ultra Imaging Photoelectron Spectrometer (Kratos Analytical Ltd., UK), using an Al Ka (hv = 1486.7 eV) X-ray source that was calibrated to the binding energy of C_1s_ (284.8 eV) by adventitious carbon. Transmission electron microscopy (TEM) images were obtained in a FEI Tecnai T20 (FEI Company, USA) transmission electron microscope at 200 kV with a point-to-point resolution of 0.35 nm. The samples were prepared by pipetting one drop of the product’s suspension onto the carbon-coated copper grid (5–50 nm in thickness); then, the solvents of the samples were removed by vacuum drying. The nanoparticle size analyses were conducted using Image J 1.34s.

## 3. Results and Discussion

### 3.1. The Photophysical Characterization and Relationships between PL Property and Size Effect of AuNC@MUA

**Figure 1:**
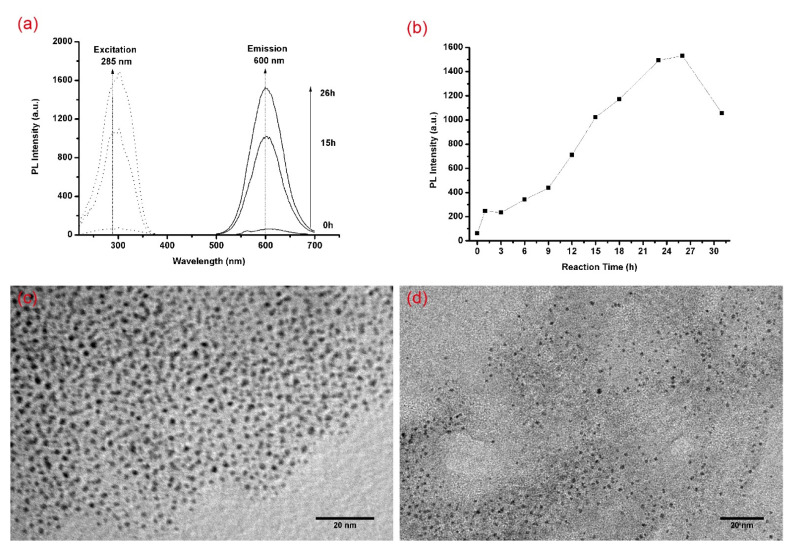
(**a**) The variations in the excitation and emission spectra; (**b**) the variations in PL intensity in the synthesis process of AuNC@MUA; (**c**) a TEM image of as-synthesized AuNP@MUA; (**d**) a TEM image of as-synthesized AuNC@MUA. The small sharp peaks at 300 nm in the excitation spectra were from the scattering of the light source.

The partial PL and PLE spectra of the etching process at different times are shown in Figure 1a. The PL spectrum at 0 h is from the AuNP@MUA, and the spectra at 15 and 26 h belongs to the formation process of the AuNC@MUA. The PL peak intensity varies at the different times shown in Figure 1b. From that, we know that 26 h was the optimal reaction time judged using PL intensity. Therefore, the etching reaction was completed at 26 h. The TEM images of the AuNP@MUA and AuNC@MUA are shown in Figure 1c,d, and the average diameters of the gold core were 2.01 ± 0.25 nm (*n* = 100) and 1.72 ± 0.22 nm (*n* = 100), respectively. The average diameter decreased by 0.29 nm after 26 h of etching. The emission peaks of the AuNP@MUA and AuNC@MUA were at 610 and 600 nm, respectively. The maximum emission wavelengths were not particularly sensitive to the diameter between 2.01 and 1.72 nm; however, the PL peak intensity improved by about 23 times. Rhodamine 6G was chosen as the reference, and the QY of AuNC@MUA was determined to be 3.4% in water (pH 9) (see the Supplementary Materials, Figure S1).

**Figure 2:**
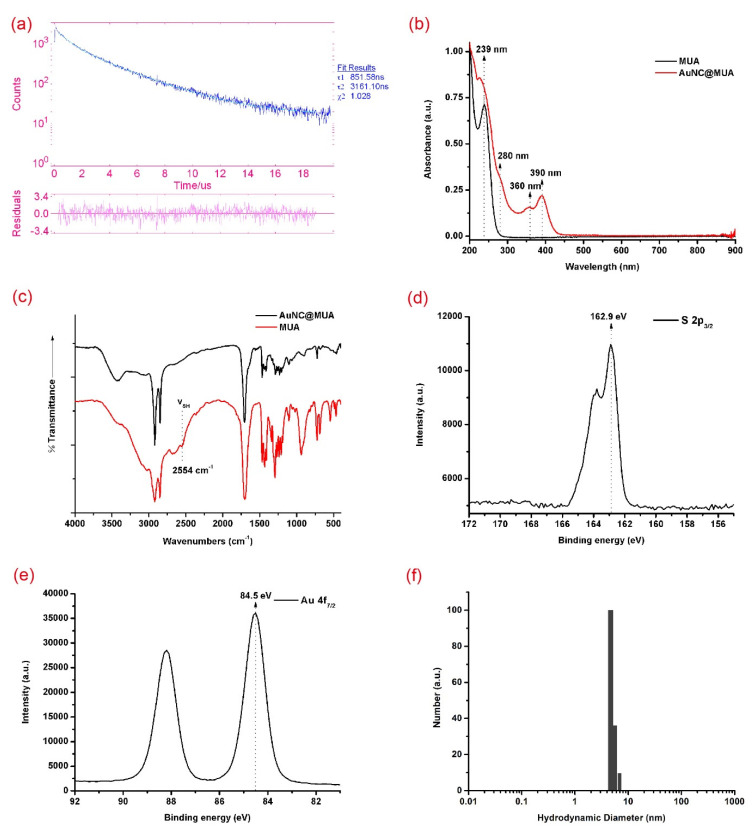
(**a**) PL lifetimes of as-synthesized AuNC@MUA; (**b**) UV-Vis absorption; (**c**) Fourier transform infrared spectra of MUA and AuNC@MUA; (**d**) S 2p; (**e**) Au 4f spectra; (**f**) dynamic light scattering of AuNC@MUA.

To study the types of PL judged by lifetimes as well as the excitation states, the PL lifetimes of the AuNC@MUA were measured. Figure 2a shows that the AuNC@MUA presented two different lifetimes at 851.58 ns (20.98%) and 3161.10 ns (79.02%). The long PL lifetimes (microseconds, μs) and large stokes-shift (>100 nm) supported that they were phosphorescent from a triplet state, instead of fluorescence. The two lifetime components suggested that there were two first excitation states. To explore the sources of emission, UV–Visible absorbance spectra of the AuNC@MUA was carried out (Figure 2b). Compared with MUA, three obvious absorption peaks appeared at 280 nm, 360 nm, and 390 nm. The absorption peak at 280 nm corresponded with the PLE peak at 285 nm. Although the AuNC@MUA at 360 nm and 390 nm also had strong absorptions, it was hardly emissive when these absorption peaks were chosen as excitation wavelengths. The sources of these absorption peaks aroused great interest. The extremely large stokes-shift (>300 nm) and the mismatch between the excitation peaks and absorption peaks of the AuNC@MUA, which were quite different from the photophysical properties of AuNCs synthesized by other researchers [17,19,29,30,31], were very suitable for studying the emission mechanism.

### 3.2. Colloidal Characterization of AuNC@MUA

To explore the interaction between gold and thiolates, Fourier transform infrared (FTIR) was first measured. In Figure 2c, except wavenumbers at 2554 cm^−1^, the peak shape and position of the MUA and AuNC@MUA have few differences. No absorption peak was present at 2554 cm^−1^ of AuNC@MUA, whereas MUA had one. This result suggested that a proton of –SH was lost [32]. To further explore the property of the Au thiol interaction and to analyze the valence states of gold in prepared AuNC@MUA, we carried out X-ray photoelectron spectroscopy (XPS) measurements. In XPS spectra (Figure 2d), the appearance of S 2p_3/2_ with a binding energy of 162.9 eV further indicated the formation of Au–S bonds [33,34], excluding the existence of disulfide (~164.0 eV) or SO^3−^ groups (~168.0 eV) [35]. The Au XPS spectra (Figure 2e) showed that the binding energy (BE) of the Au 4f_7/2_ of AuNC@MUA was located between the Au(0) BE (84.0 eV) of bulk gold and the Au(I) BE (86.0 eV) of gold thiolate [15], suggesting the coexistence of Au(0) and Au(I) in the luminescent AuNC@MUA. To study whether there was a Au(I)-thiolate polymers formation in addition to TEM, dynamic light scattering (DLS) was carried out. The hydrodynamic diameters of AuNC@MUA were 5.1 ± 0.66 nm (Figure 2f). These two results indicated that there were no giant polymers.

### 3.3. Source of Absorption and Emission Mechanisms of AuNC@MUA

These absorption peaks mainly came from two aspects; one was caused by the gold core, and the other was caused by the gold and ligand interactions. Therefore, if we could partially bring the ligands down from the gold core on the condition that they did not influence the size and valence state of the surface gold atoms, we theorized that the absorption caused by the gold and ligand interactions would decrease, while absorption caused by the gold core would increase, because the impediments of the electron transitions arising from the ligands would be reduced. A small amount of NaClO solution was used to partially bring down the ligands, because thiols or disulfides can be oxidized to sulfonyl chlorides using NaClO [36]. Moreover, the standard oxidation–reduction potential of ClO^−^/Cl^−^ is lower than that of Au(Ⅲ)/Au(Ⅰ) [37], so the valence state of the surface gold atoms were not changed by NaClO.

**Figure 3:**
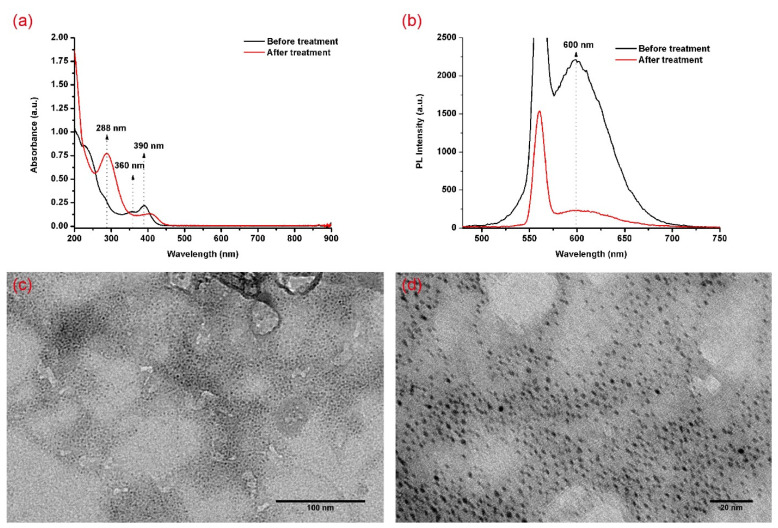
(**a**) The absorption and (**b**) emission spectra of AuNC@MUA before and after NaClO aqueous solution treatment; (**c**) and (**d**) TEM images of AuNC@MUA after NaClO aqueous solution treatment under different magnifications.

The variation in the absorption spectra after the treatment of AuNC@MUA using NaClO is shown in Figure 3a. We found that no obvious absorbance over 500 nm was presented, which meant that plasmonic-gold nanoparticles did not form. To further confirm the absence of large particles, the TEM images (Figure 3c,d) were received. After the treatment, the average diameter of the gold core was 1.71 ± 0.22 nm (*n* = 100). No statistical differences were found compared with the average diameter of the gold core before treatment (1.72 ± 0.22 nm, *n* = 100). Under this condition, we can subsequently discussed the variations in absorption spectra.

**Figure 4:**
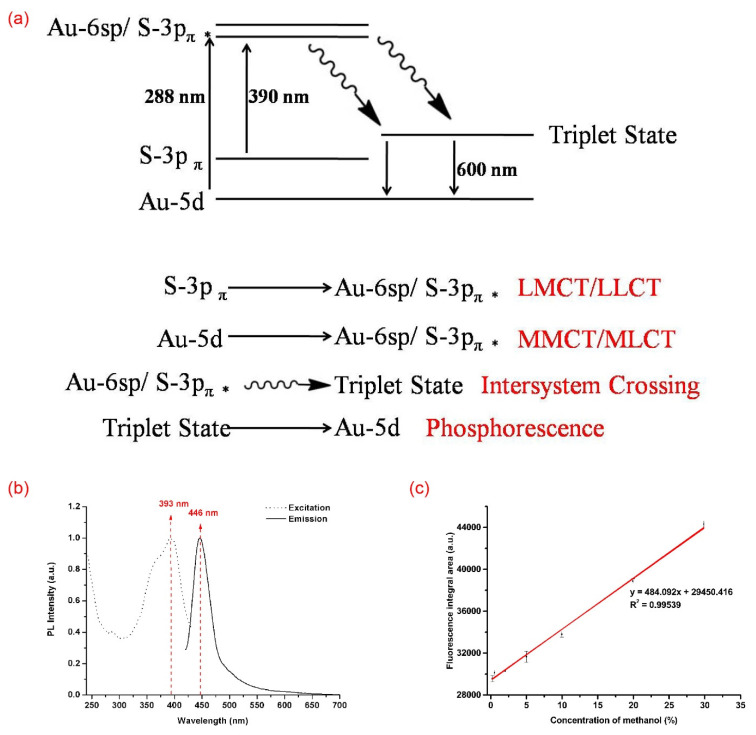
(**a**) Proposed emission mechanism of AuNC@MUA; (**b**) excitation and emission of AuNC@MHA; (**c**) linear correlation between the concentration of methanol and the PL integral area. LMCT, ligand-to-metal charge transfer; LLCT, ligand-to-ligand charge transfer; MMCT, metal-to-metal charge transfer; MLCT, metal-to-ligand charge transfer.

In Figure 3a, the absorption peak at 288 nm displays a remarkable increase, whereas the absorption peaks at 360 nm and 390 nm display declines. Hence, we reached the following conclusion that was anticipated above: the former mainly came from the gold core, while the latter mainly came from gold and ligand interactions. As the PLE peak at 285 nm coincided with the absorption peak at 288 nm, the emission of the AuNC@MUA was derived from the gold core. A prominent decline in the emission was found after the NaClO aqueous solution treatment was added (Figure 3b), suggesting that the emission mainly came from the surface gold atoms of the gold core, because the PL intensity was very sensitive to the surroundings. Based on these results and related references [22,38,39,40], we proposed an emission mechanism of AuNC@MUA (Figure 4a); the excitation at 288 nm leads to the excitation of Au-5d orbital electrons into the Au-6sp orbitals and S-3p_π__∗_ orbitals through intersystem crossing (ISC), to the lowest metal-centered excited triplet state. Moreover, radiative recombination is followed by radiative relaxation to the Au-5d orbitals and by phosphorescence (600 nm).

### 3.4. Regulation of Emission Mechanism

To further explore the relationship between the emission mechanism and gold nanocluster size, MHA, which has a stronger etching ability and a similar structure to MUA, was selected for the experiments. Under the same experimental conditions, the PL and PLE spectrum variations in the synthesis process of AuNC@MHA are shown in Figure 4b. Compared with AuNC@MUA, $\lambda_{\max}^{\mathrm{ex}}$ of AuNC@MHA changed from 285 nm to 393 nm, and $\lambda_{\max}^{\mathrm{em}}$ changed from 600 nm to 446 nm. The excitation peak at 393 nm corresponded with an absorption peak at 390 nm (Figure S2), which was mainly derived from LMCT, proven thereinbefore. In addition, measured by TEM, the gold core size of AuNC@MHA was 1.62 ± 0.23 nm (*n* = 100) (Figure S3), which was smaller than that of AuNC@MUA. Whether the ligand or cluster size regulated the emission mechanism remains to be answered. As this reaction was extremely fast, we could not observe the intermediate process of the PL variations. Therefore, AuNC@MHA_im_ was synthesized using a quarter of MHA in the synthesis of AuNC@MHA_im_. The PL variations were the same as that of AuNC@MUA (Figure S4). With the etching reaction by inadequate MHA, the intensity of the emission peak at 600 nm gradually increased. No emission was found when excited at 390 nm (data not shown), which indicated that the emission mechanism switched by size instead of ligand. According to the varied characteristics of the PL and PLE spectra, we speculated that when the sizes changed, the density of the free electrons of the gold core, the density of the Au–S bonds, and the Au–Au interactions all played important roles in PL, which subsequently also changed. Moreover, they affected the triplet state, leading to different emission mechanisms [12,19,38].

### 3.5. Detecting Methanol in Adulterated Wine Model

Methanol is mainly used in the chemical industry; however, sometimes it is mixed with ethanol in fake wine to increase the profit. The morbidity and mortality from methanol remain high, even with effective treatment. Methanol poisoning affects people not only in developing countries, but in developed countries also [41,42]. Therefore, developing a facile method to detect methanol is essential. After learning that AuNCs have different PL intensities in methanol and water through related references [18,43] and experiments (Table S1 and Figure S5), we performed a number of experiments to detect methanol in a simplified adulterated wine model (methanol-ethanol-water mixtures). The total volume of methanol and ethanol was fixed at 500 μL (V_methanol_ + V_ethanol_ = 500 μL) by adjusting the concentration of methanol to observe the variations in the PL integral area. As a small amount of methanol was present in the ethanol reagent, we recalculated the volume and concentration of methanol (Table S2). The three measurements of the PL integral area and the average value with different concentrations of methanol are shown in Table S3. We found that the correlation between the concentration of methanol and the PL integral area was quite linear (Figure 4c). The limit for the detection of methanol could reach 0.224% in the methanol-ethanol-water mixtures using this method, which was a simple and easy-to-use method compared with potassium permanganate oxidation [44]. Therefore, AuNC@MUA may potentially be used in detecting methanol in fake alcohol. The methanol detection mechanism is as follows: the PL intensity of the AuNCs was closely related to the polarity of the surface ligands and solvent environment [27,28,45]. The ligand of the AuNC, the MUA was covalently bound to the gold core via sulfhydryl group and exposed carboxyl groups on the surface. The polarity value of the carboxylic acid was about 6.0. As solvents, the polarity values of methanol, ethanol and water were 5.1, 4.3 and 10.2 respectively [46]. Based on the theory of “similarity-intermiscibility”, the carboxyl-capped AuNC@MUA tended to be dispersed in the methanol, followed by ethanol and finally the water. An excellent dispersibility in methanol was conducive to higher PL intensities. On the contrary, AuNC@MUA were more likely to accumulate in water and collide to cause energy loss, resulting in a decrease in PL intensities.

## 4. Conclusions

In summary, the luminescent AuNC@MUA was synthesized and characterized. A method of oxidizing the ligands that protected the core of the AuNCs was used to reveal the sources of emission; on the basis of this discovery and efforts by other groups, the emission mechanism of the AuNC@MUA was illuminated. By changing the stronger etching ability ligands, the emission mechanism was regulated by changing the size of the gold nanocluster. On the basis of the experimental results, methanol in the simplified adulterated wine model was detected. The correlation between the concentration of methanol and fluorescence integral area was quite linear, which indicated that AuNC@MUA could potentially be used for detecting methanol in fake alcohol.

## Data Availability

The data presented in this study are available from the corresponding author upon reasonable request.

## Supplementary Materials

The following are available online at https://www.mdpi.com/article/10.3390/ma14216342/s1, Figure S1: Measurement of QY for AuNC@MUA, Figure S2: Absorption spectrum of as-synthesized AuNC@MHA, Figure S3: TEM image of as-synthesized AuNC@MHA, Figure S4: PL variations of AuNC@MHA_im_, in which the sharp peak at 560 nm was from the scattering of the light source, Figure S5: PL intensities under different solvents according to Table S1, Table S1: AuNC@MUA solution dispersed in water, methanol, and ethanol, Table S2: Recalculated volume and concentration of methanol according to the methanol and ethanol reagents used in this study, Table S3: The PL integral area, the average value, and standard deviation under different concentrations of methanol measure three times.
