# Supplementary material for: The Emission Mechanism of Gold Nanoclusters Capped with 11-Mercaptoundecanoic Acid, and the Detection of Methanol in Adulterated Wine Model
Source: Materials (Basel). 2021 Oct 23;14(21):6342. doi: 10.3390/ma14216342 (PMC8585185; doi:10.3390/ma14216342)
Supplement: Supplementary file 1 [file materials-14-06342-s001.zip › materials-1408178-supplementary.pdf]

materials

Supplementary Materials

The Emission Mechanism of Gold Nanoclusters Capped with 11-Mercaptoundecanoic Acid, and the Detection of Methanol in Adulterated Wine Model

Ming Wei [1], Ye Tian [1], Lijun Wang [1], Yuankai Hong [1,*], Dan Luo [2,*] and Yinlin Sha [1,*]

[1] Single-Molecule and Nanobiology Laboratory, Department of Biophysics, School of Basic Medical Sciences, Peking University, Beijing 100191, China; weiming@bjmu.edu.cn (M.W.); ye.tian1@ge.com (Y.T.); lijunwang@bjmu.edu.cn (L.W.)
[2] CAS Center for Excellence in Nanoscience, Beijing Key Laboratory of Micro-nano Energy and Sensor, Beijing Institute of Nanoenergy and Nanosystems, Chinese Academy of Sciences, Beijing 100083, China
* Correspondence: ykhong@bjmu.edu.cn (Y.H.); luodan@binn.cas.cn (D.L.); shyl@hsc.pku.edu.cn (Y.S.)

Citation: Wei, M.; Tian, Y.; Wang, L.; Hong, Y.; Luo, D.; Sha, Y. The Emission Mechanism of Gold Nanoclusters Capped with 11-Mercaptoundecanoic Acid, and the Detection of Methanol in Adulterated Wine Model. *Materials* **2021**, *14*, 6342. https://doi.org/10.3390/ma14216342

Academic Editors: Mikhael Bechelany and Daniela Iannazzo

Received: 20 September 2021
Accepted: 20 October 2021
Published: 23 October 2021

Publisher's Note: MDPI stays neutral with regard to jurisdictional claims in published maps and institutional affiliations.

Copyright: © 2021 by the authors. Submitted for possible open access publication under the terms and conditions of the Creative Commons Attribution (CC BY) license (http://creativecommons.org/licenses/by/4.0/).

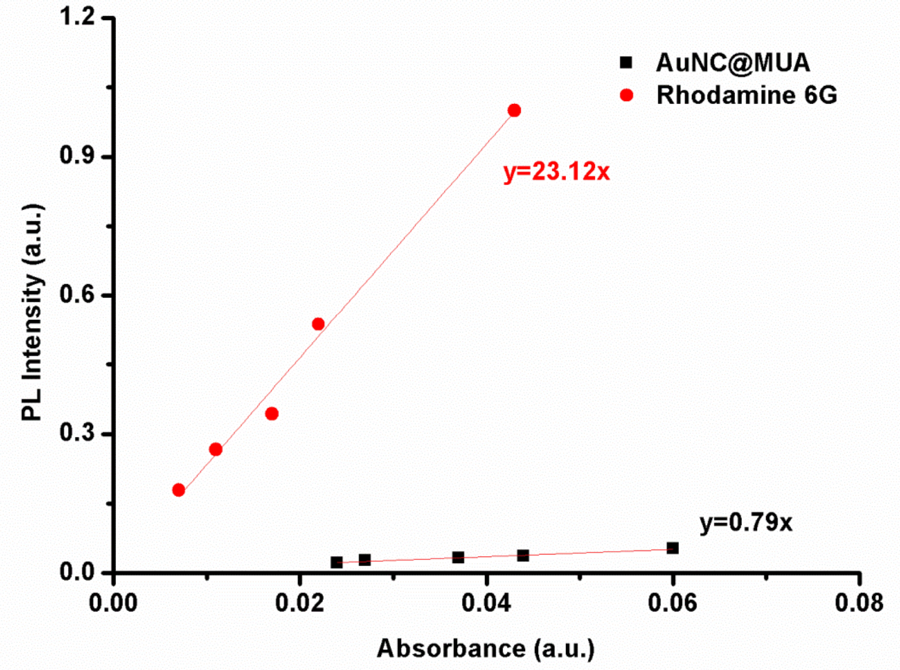

Figure S1. measurement of QY of AuNC@MUA.

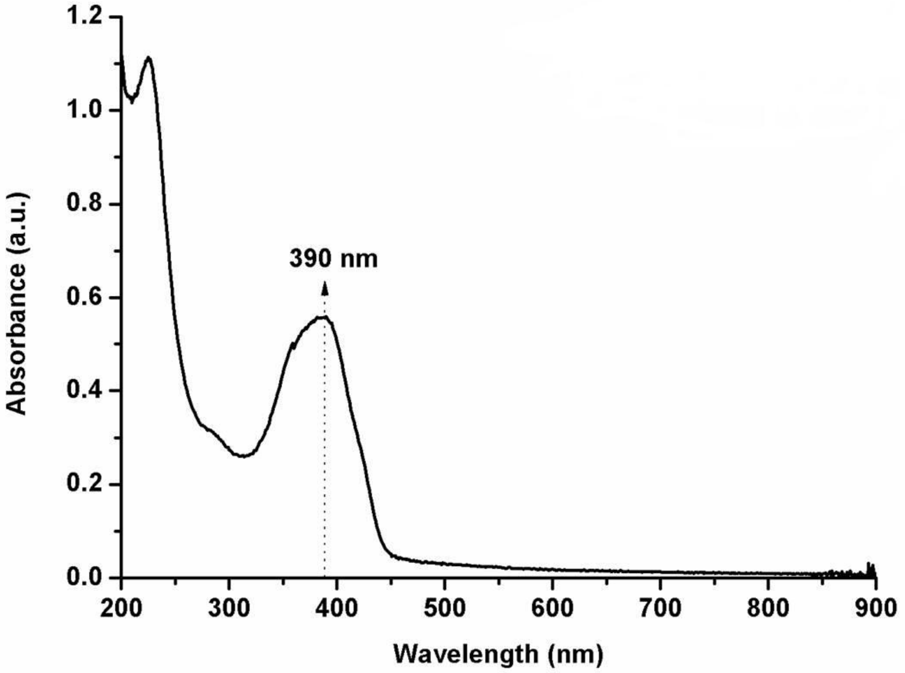

Figure S2. absorption spectrum of as-synthesized AuNC@MHA.

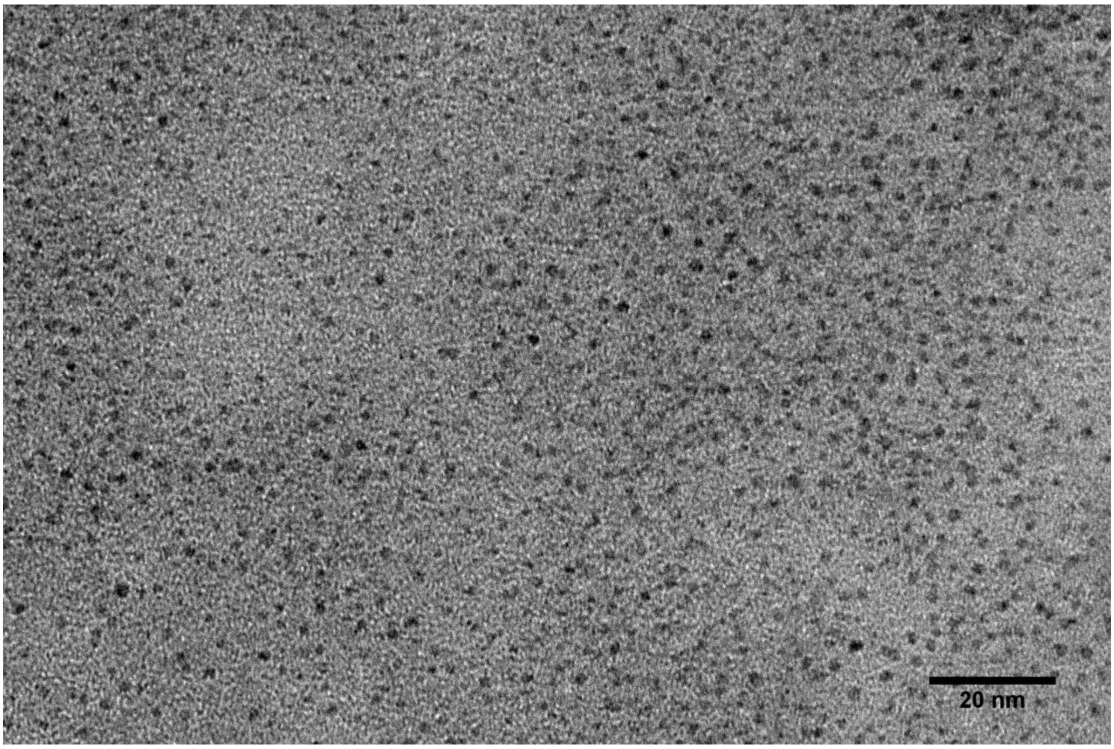

Figure S3. TEM image of as-synthesized AuNC@MHA.

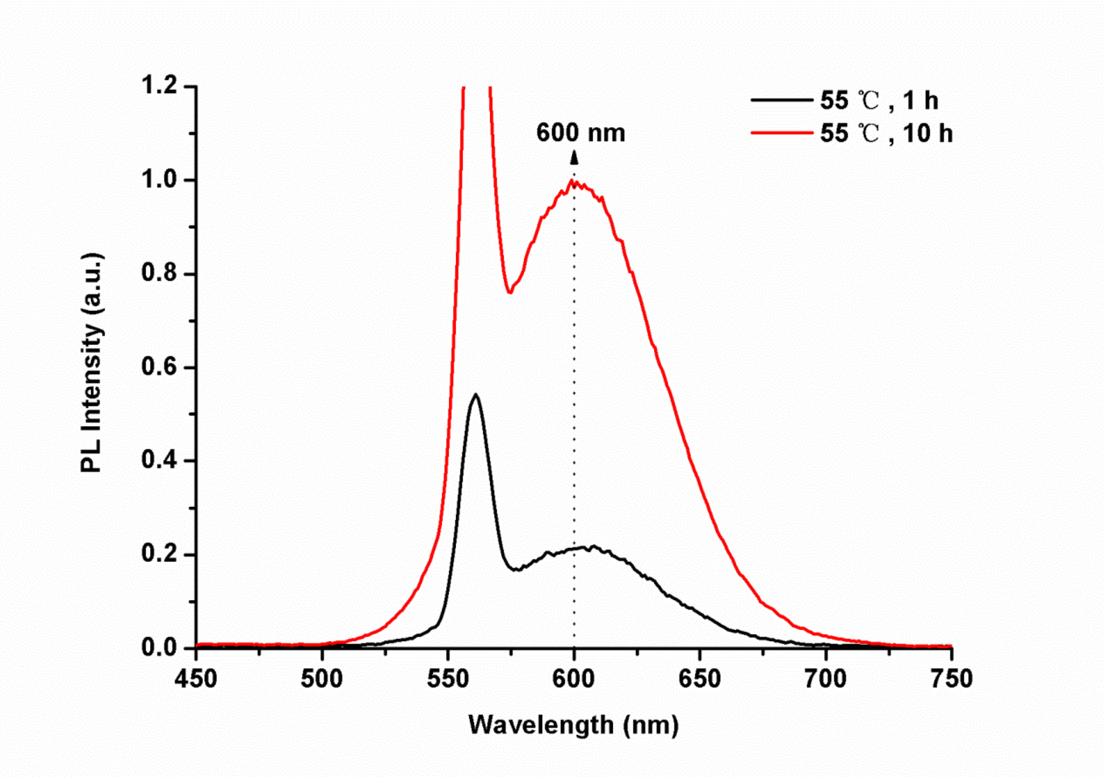

Figure S4. PL variations of AuNC@MHA_{im}, the sharp peak at 560 nm was from the scattering of the light source.

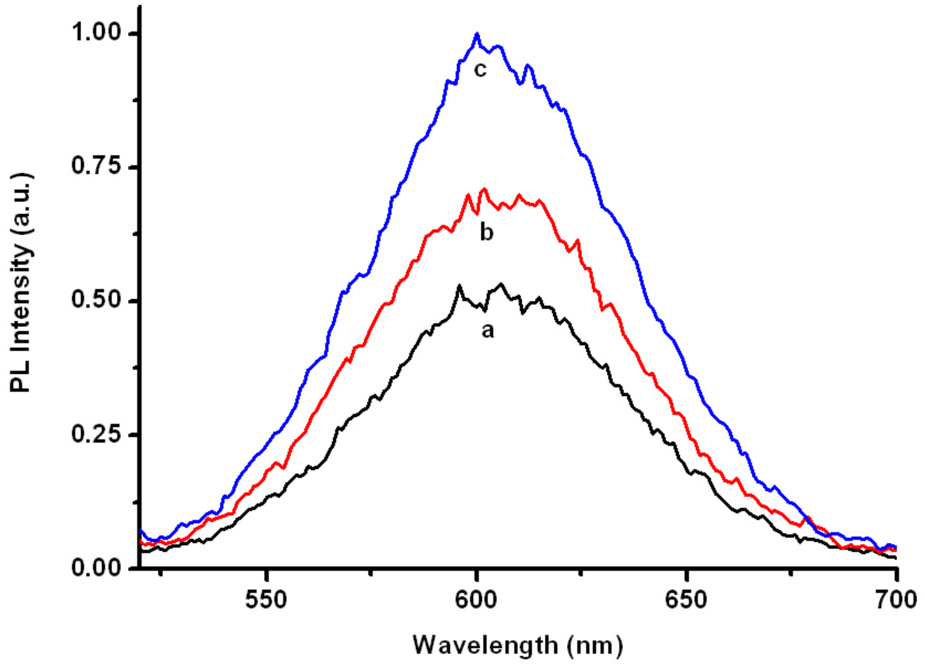

Figure S5 PL intensities under different solvents according to Table S1.

Table S1. AuNC@MUA solution dispersed in water, methanol and ethanol.

**Unit (μL)**	**a**	**b**	**c**
AuNC@MUA	100	100	100
Methanol	0	0	500
Ethanol	0	500	0
Ultrapure water	900	400	400

Table S2 Recalculated volume and concentration of methanol according to the methanol and ethanol reagents used in this study.

Unit (μL)	A	B	C	D	E	F	G
AuNC@MUA	500	500	500	500	500	500	500
Methanol	2	5	20	50	100	200	300
Ethanol	498	495	480	450	400	300	200
Total volume of methanol	2.24	5.22	20.14	49.98	99.70	199.15	298.60
Concentration of ethanol (%, v/v)	49.776	49.478	47.986	45.002	40.030	30.085	20.140
Concentration of methanol (%,v/v)	0.224	0.522	2.014	4.998	9.970	19.915	29.860

Table S3 The PL integral area, the average value, and standard deviation under different concentrations of methanol measure three times.

Concentration of methanol (%, v/v)	0.224	0.522	2.014	4.998	9.970	19.915	29.860
PL integral area 1	29176.75	29903.28	30616.88	30905.03	33413.48	38622.26	44569.67
PL integral area 2	29735.00	30071.32	30258.05	32017.42	33998.03	39129.52	43932.59
PL integral area 3	29813.75	30452.69	30047.84	32069.56	33987.74	38923.12	44481.75
Average value	29575.50	30142.43	30307.59	31664.01	33799.75	38891.63	44328.00
Standard deviation	283.74	229.86	234.94	537.10	273.17	208.28	281.89
